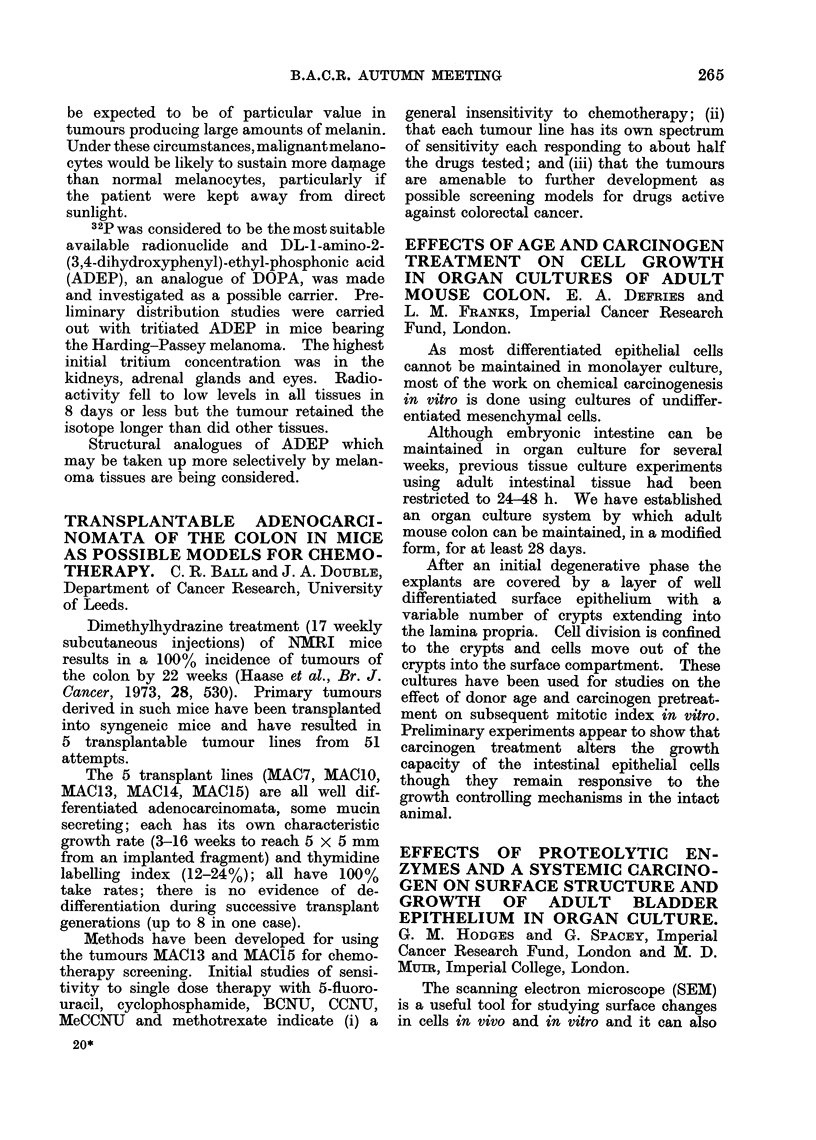# Proceedings: Transplantable adenocarcinomata of the colon in mice as possible models for chemotherapy.

**DOI:** 10.1038/bjc.1975.53

**Published:** 1975-02

**Authors:** C. R. Ball, J. A. Double


### TRANSPLANTABLE ADENOCARCINOMATA OF THE COLON IN MICE AS POSSIBLE MODELS FOR CHEMOTHERAPY.

C. R. Ball and J. A. Double, Department of Cancer Research, University of Leeds.

Dimethylhydrazine treatment (17 weekly subcutaneous injections) of NMRI mice results in a 100% incidence of tumours of the colon by 22 weeks (Haase *et al.*, *Br. J. Cancer*, 1973, **28**, 530). Primary tumours derived in such mice have been transplanted into syngeneic mice and have resulted in 5 transplantable tumour lines from 51 attempts.

The 5 transplant lines (MAC7, MAC10, MAC13, MAC14, MAC15) are all well differentiated adenocarcinomata, some mucin secreting; each has its own characteristic growth rate (3–16 weeks to reach $5 \times 5$ mm from an implanted fragment) and thymidine labelling index (12–24%); all have 100% take rates; there is no evidence of dedifferentiation during successive transplant generations (up to 8 in one case).

Methods have been developed for using the tumours MAC13 and MAC15 for chemotherapy screening. Initial studies of sensitivity to single dose therapy with 5-fluorouracil, cyclophosphamide, BCNU, CCNU, MeCCNU and methotrexate indicate (i) a general insensitivity to chemotherapy; (ii) that each tumour line has its own spectrum of sensitivity each responding to about half the drugs tested; and (iii) that the tumours are amenable to further development as possible screening models for drugs active against colorectal cancer.